# Acceptable or Not: An In-depth Analysis of Adolescent Competitive Athletes’ Perceptions on Abusive Coaching Behaviors

**DOI:** 10.1177/08862605241303958

**Published:** 2024-12-30

**Authors:** Élise Marsollier, Denis Hauw, Fabienne Crettaz von Roten

**Affiliations:** 1 Université de Lausanne, Switzerland

**Keywords:** child abuse, community violence, neglect, physical abuse, prevention of child abuse

## Abstract

The present study aimed to conduct an in-depth analysis of adolescent competitive athletes’ perceptions on abusive coaching behaviors. Our aims were thus to (a) identify the acceptable abusive coaching behaviors and (b) characterize qualitatively the criteria for the acceptance of abusive coaching behaviors. Based on the study goal, an Abusive Coaching Behavior Grid was developed and completed by 356 French-speaking athletes, among which 10 were interviewed to justify where they draw the line between acceptable and unacceptable coaching behaviors. Quantitative analysis showed that shaking, shouting at, or asking athletes to perform until exhaustion were considered acceptable. Quebec and female athletes tended to accept fewer different abusive behaviors, but there were no differences by sport characteristics. The perception on abusive coaching behaviors was influenced by expectations about the coaching role, negative effects of coaching behaviors, circumstances in which the behaviors occur, and the nature of behaviors. The present study raises the importance of questioning cognitive schemas shared by groups of athletes as well as the norms coaches convey and the behaviors they adopt.

Participation in sports at a competitive level requires sacrifice, perseverance, and continual self-improvement, with coaches adopting those behaviors that promote high performance (Preston & Fraser-Thomas, 2018). Since several forms of abuse have been reported, particularly by adolescent competitive athletes (e.g., Marsollier et al., 2021; Pinheiro et al., 2014),there is a need to understand why such high prevalence rate could be observed. For example, the prevalence rate of the psychological abuse of competitive athletes varied from 60% to 87%, and the rate of physical abuse ranged from 14% to 53% (e.g., Hauw et al., 2024b; Marsollier et al., 2021; Willson et al., 2022). While coaches have been one of the most cited perpetrators of these two forms of abuse (e.g., Marsollier et al., 2021; Willson et al., 2022), a better understanding of how these abusive coaching behaviors can exist is needed.

Psychological abuse is defined as “a pattern of deliberate non-contact behaviors by a person within a critical relationship role that has the potential to be harmful” (Stirling & Kerr, 2008a, p. 178). Qualitative studies with former athletes have revealed that the primary psychologically abusive behaviors that coaches inflict are shouting (Gervis & Dunn, 2004; Stirling & Kerr, 2008b), controlling weight (Pinheiro et al., 2014), criticizing (Stirling & Kerr, 2008b), denigrating, threatening, and even humiliating (Gervis & Dunn, 2004). Physical abuse is considered a non-accidental trauma or physical injury caused by punching, beating, kicking, biting, burning, or otherwise harming an athlete (Mountjoy et al., 2016, p. 1021). The few studies that have examined this specific form of sports-related abuse have revealed that the primary physically abusive behaviors that coaches inflict are forcing athletes to train or compete while injured or exhausted, training/competing with injuries, and corporal punishment such as pushing, shoving, shaking, slapping, kicking, or throwing an object at the athlete (McPherson et al., 2017; Pinheiro et al., 2014).

Beyond prevalence rates, researchers sought to identify factors associated with the risk of athletes’ victimization for interpersonal violence (i.e., individual, relational, organizational, and sociocultural factors). In the perspective of individual factors, male athletes have been described to be more at risk of physical abuse than female athletes (Hauw et al., 2024a; Parent & Vaillancourt-Morel, 2020), while international-level athletes appear more at risk to experience both psychological and physical abuse (Vertommen et al., 2016; Willson et al., 2022). Regarding the type of sport practiced, team sports and individual sports athletes reported more physical abuse than opposition or endurance sport (Hauw et al., 2024a; Parent & Vaillancourt-Morel, 2020). Regarding the sociocultural mechanisms explaining the phenomenon of violence in sport, Parent and Fortier (2018) listed the notions of normalization, tolerance, and acceptance as risk factors for athletes’ victimization. In fact, while athletes are increasingly more likely to denounce coaching abuse in the media, several processes have been suggested to explain why it remains acceptable to athletes. The first is conformity to a sports ethic conceptualized as “a set of norms accepted as the dominant criteria for defining what it means, in their social worlds, to be defined and accepted as an athlete in power and performance sports” (Coakley & Donnelly, 2009, p. 155). According to Coker-Cranney et al. (2018), the process by which conformity to the sports ethic norms develops among athletes is gradual and starts early in their careers. Throughout their athletic development, many athletes do not question these norms and conform to them (Smits et al., 2017), which lead them to accept psychological and physical coaching abuses (e.g., Marsollier & Hauw, 2022; Pinheiro et al., 2014). A second process is the normalizing cognition, also known as moral disengagement, which includes the reconstruction of the harmful behavior by using moral justification, minimizing, ignoring, or misconstruing the consequences of violence (Bandura et al., 1996). Normalizing cognition appears to mediate the exposure to coaches’ abusive behaviors and the affective and behavioral effects of the exposure, leading athletes not to question the abusive behavior. Interestingly, research in the field of family violence may also be informative for understanding athletes’ acceptance of abuse. A key point is the perspective of individual development, where the acceptance of parent–child aggression is a risk factor of child abuse (Gracia et al., 2020). In order to explain abuse acceptance, researchers in this field refer to the social information processing model of aggressive behavior (Huesmann, 1988; Milner, 2000). According to Gracia et al. (2020), the social information processing model shows how cognitive schemas, including the acceptance of violence in close relationships (e.g., approval of parent–child aggression), influence how people interpret and ultimately respond to conflictual situations (e.g., discipline strategies with children). Based on this model, sports-related abuse acceptance could be grounded in a personal history of aggression or domination that may have led to attitudes of acceptance in a range of physical and psychological abuse situations, including coach-athlete relationships. The elite sports culture might then be viewed as affording contextually shared cognitive schemas related to interpersonal violence that explain the acceptance of coaches’ abusive behaviors.

Thus, many factors are associated with the risk of athletes’ victimization of interpersonal violence, including individual factors (gender, type of sport) and sociocultural factors, such as acceptance. While athletes may accept many forms of physical and psychological abuse due to various socialization processes that led them to normalize and legitimate such behaviors, there is a need to describe and explain the extent to which certain abusive behaviors may be deemed acceptable by athletes. Since interpersonal violence prevalence rates mainly concern adolescent athletes, and acceptance is built up over the course of a sporting career, its description from adolescent athletes’ perspectives appears essential.

This study was conducted within a Francophone context that emphasized specific cultural considerations for sport participation. In the Francophonie, sport is closely tied to public organization and focuses on educational values, such as doping prevention, inclusion, and social learning, with less emphasis on performance in contrast to Anglo-Saxon cultures (Mouiller, 2023). French-speaking adolescent athletes living in Europe could be considered representative of broader adolescents’ populations in these countries in certain aspects. They embody the culture of rapid success but are also prone to rapidly disengage from their activities when problems and costs arise. Furthermore, they are affected by an increased prevalence of anxious-depressive symptoms (Haut Conseil de la Famille de l’Enfance et de l’Age, 2023). Athletes can sometimes assume some differences with the sense of being exceptional even if the culture of merit does not favor it before they achieved multiple successes. In a recent study, they have also been shown as more vigilant, self-centered, cautious, and meticulous than the general population (Hauw et al., 2024b).

This study therefore aimed to conduct an in-depth analysis of adolescent competitive athletes' perceptions on abusive coaching behaviors. Our aims were thus to (a) identify the acceptable abusive coaching behaviors and (b) characterize qualitatively the criteria for the acceptance of abusive coaching behaviors.

## Methods

### Research Design

This study used a mixed methodology defined as a method adopting both quantitative and qualitative designs within the same study (Caruth, 2013). We used an “explanatory sequential” design consisting of first collecting quantitative data and then enriching it with qualitative data (Caruth, 2013). First, we collected quantitative data with an online questionnaire on the abusive behaviors potentially used by coaches with their young athletes. Based on the answers, semi-structured interviews were conducted to elicit explanations and justifications for the questionnaire responses. The quantitative data allowed to identify the acceptable abusive coaching behaviors according to individual factors (gender, country, and type of sport), whereas the qualitative data characterized the criteria of acceptance of these abusive behaviors.

### Recruitment of Participants

After receiving the approval of the (hidden for anonymity) ethics committee (hidden for anonymity), we recruited athletes who (a) were between 12 and 18 years old, (b) practiced their sport in competition, (c) lived or played in Quebec, France or Switzerland, and (d) spoke French fluently. For the quantitative part, the description of the study was sent by e-mail to the extended sports network in which two of the three authors have been active for several years. Also, sports club presidents and coaches whose contact information was available on the internet were sent an e-mail of explanation or called by phone. In parallel, the study has been promoted on several social media (e.g., LinkedIn, Instagram, and Facebook). The online questionnaire was e-mailed to anyone wishing to take part in the study, once the consent form had been signed by a parent for athletes under 16. For the qualitative part, the athletes were recruited among the volunteers until no new data emerged. Specifically, the respondents had the opportunity at the end of the questionnaire to share their e-mail address in order to be contacted again for an interview according to their availability.

### Data Collection

Data collection took place from March to June 2021.Two tools were developed to collect the data: a questionnaire including the Abusive Coaching Behavior Grid for quantitative data and a cognitive interview guide for qualitative data.

### Abusive Coaching Behavior Grid

The Abusive Coaching Behavior Grid was developed from existing coaching abuse classifications (Fortier et al., 2020; Stirling, 2009) and current measurement tools for physical and psychological abuse (Interpersonal Violence against Children in Sport, IViS, Vertommen et al., 2016; Violence Toward Athletes Questionnaire, VTAQ-C, Parent et al., 2019). An initial list of 49 abusive behaviors was first organized into two main categories: physical abuse behaviors (13 items) and psychological abuse behaviors (36 items). Then, drawing inspiration from Stirling’s classification (2009), psychological abuse was separated into verbally abusive behavior (13 items), non-verbally abusive behavior (11 items), and demands on the athlete (12 items). The final version of the Abusive Coaching Behavior Grid was organized into four main categories: (a) contact behaviors (e.g., shoving/pushing the athlete), (b) verbal behaviors (e.g., yelling at the athlete), (c) control behaviors (e.g., punishing the athlete for poor performance), and (d) demands (e.g., asking the athlete to compete while injured). In line with studies examining family violence (Gracia et al., 2020), for each category, participants were asked to rate the behaviors on a 6-point Likert scale from 1: “completely acceptable behavior” to 6: “completely unacceptable behavior” while having the possibility of specifying “Don’t know” (DK). Unless otherwise specified, they were asked to reflect on the behaviors as being adopted by a coach toward an athlete, face-to-face and without the presence of witnesses to highlight the seriousness in the behaviors. The behaviors to be evaluated were presented in a neutral way, as “behaviors that can be observed in coaches.” As a preamble to the grid, participants were asked to complete sociodemographic questions (e.g., country of residence, sex at birth, age, sport, etc.). The grid was created on the Lime Survey platform to allow for online administration, making recruitment and administration easier. The grid took about 15 min to complete (*M* = 14.29 min; *SD* = 9.48). At the end of it, athletes interested in discussing their answers during an interview were invited to add their email address.

### Interview Guide

As the purpose of the cognitive interviews was to collect additional verbal information about the Abusive Coaching Behavior Grid (Beatty & Willis, 2007), it was organized into two sections: (a) justifications of Abusive Coaching Behavior Grid scores, and (b) characterization of the boundary between the acceptableness and unacceptableness of each coaching behavior. First, a few initial questions were asked in order to establish a relationship of trust with the participants (e.g., How many years have you been practicing your sport?; What made you choose this sport?). Second, based on the Abusive Coaching Behavior Grid scores, the participants were asked to justify their rating for each behavior (e.g., In the contact category, you rated all the coaching behaviors as totally unacceptable (6), could you explain why?; You rated the item of belittling or ridiculing the athlete as a 5, so it’s a tiny bit less unacceptable than the other three. Could you justify or explain why?). Finally, for each category of abusive coaching behavior (i.e., contact, verbal, control, demand), participants were asked to describe the point at which these coaching behaviors become unacceptable (e.g., In this category, where would you draw the line between acceptable and unacceptable coaching behaviors?). Before each interview, the participants received a summary table containing their answers to the questionnaire to enable them to prepare for the discussion. Due to the COVID-19 health situation, each interview was conducted via Zoom, with video and audio both recorded after each participant agreed. In order to ensure the rigor and consistency of our methodology, a pilot interview was conducted with a young swimmer. Two changes were made to facilitate the qualitative data collection: a simplification of the wording of questions for a young audience and the summary table formatting.

### Participants of the Quantitative Research

A total of 356 athletes began to respond to the research questionnaire, and one respondent only answered questions in the sociodemographic section but did not start the Abusive Coaching Behavior Grid. Therefore, the sample size was 355. A few subjects dropped out after the sociodemographic part and the physical coaching behavior questions, as is usual with long questionnaires: from 355 at the beginning to 329 at the end, for a loss of 7%, which is reasonable. (Added to this are DKs on items that increase as you progress through the questionnaire). Table 1 indicates that the sample median age was 15 years old, with 45% female. The distribution of age differed across genders. Most of the respondents lived in Switzerland (59%) and played team sports (79%). More than half practiced their sport at the regional/provincial level (62%) and a third at the national level (31%).

**Table 1.** Sociodemographic and Sports Characteristics of the Study Sample and Test of Distribution Across Gender.

	Total Sample (*N* = 355) *N* (%)	Female (*N* = 161) *N* (%)	Male (*N* = 194) *N* (%)	Test *p*
Age				.0024
12	17 (4.5)	15 (9.2)	2 (1)	
13	44 (12.4)	19 (11.7)	25 (12.9)	
14	72 (20.3)	22 (13.6)	50 (25.8)	
15	61 (17.2)	33 (20.4)	28 (14.4)	
16	70 (19.7)	32 (19.8)	38 (19.6)	
17	59 (16.6)	28 (17.3)	31 (16.0)	
18 and older	33 (9.3)	13 (8.0)	20 (10.3)	
Country				.0001
France	70 (19.7)	16 (9.9)	54 (27.8)	
Quebec	76 (21.4)	35 (21.7)	41 (21.1)	
Switzerland	209 (58.9)	110 (68.3)	99 (51.0)	
Type of sport				.639
Individual	73 (20.5)	35 (21.6)	38 (19.6)	
Team	283 (79.5)	127 (78.4)	156 (80.4)	
Practice level				.0010
Regional/Provincial	219 (61.8)	91 (56.5)	128 (66.0)	
National	110 (31.0)	64 (39.8)	46 (23.7)	
International	26 (7.3)	6 (3.7)	20 (10.3)	

### Participants of the Qualitative Research

Ten athletes agreed to explain their responses on the Abusive Coaching Behavior Grid. Interviews lasted between 38 and 76 min, for an average of 68 min. The athletes who represented the three countries (three Canadian, three French, four Swiss) were between 14 and 18 years old and had progressed in seven sports (tennis, hockey, badminton, swimming, diving, trampoline, artistic swimming) at a national or international level.

### Data Analysis

#### Statistical Analysis

Descriptive statistics were calculated for each sociodemographic and coaching behavior variables. Chi-square independence tests were performed to assess the differences in sociodemographic variables by gender. The structure of the Abusive Coaching Behavior Grid was assessed by a confirmatory factor analysis (available on request). An analysis was designed to mathematically quantify the acceptability and unacceptability of coaches’ behavior: below 3.5, that is, the midscore of the 6-point Likert scale, behavior was considered acceptable, when score exceeded 3.5, the behavior shifted to unacceptable. As the scores of each coaching behavior did not follow a normal distribution, we assessed the acceptance of each behavior with the one-sample Wilcoxon signed rank test (alternative hypothesis: unacceptance = score greater than 3.5). For a power of .8, a type I error of .05, an effect size of 0.2 (small), the Wilcoxon signed rank test needs at least 164 subjects. Then, the number of behaviors described as acceptable (i.e., score <3.5) in each category of behaviors was calculated for each respondent. The level of acceptance of a category of behaviors measured by this score was then compared among subgroups defined by gender, type of sport, and level of sport with a non-parametric test due to non-normality (Mann–Whitney test for gender and type of sport, Kruskal–Wallis for level of sport and country). In cases of a significant difference across level of sport or country, multiple comparisons were performed (Dwass–Steel–Critchlow–Fligner method). Practically, we considered *p*-values less than .05 to be statistically significant. The analyses were conducted with Jamovi software (version 1.6.23).

#### Thematic Content Analysis and Quality Standards

In addition to the quantitative analysis, which indicated the numerical value of what was considered acceptable or unacceptable (completely acceptable: 1, acceptable: 2, rather acceptable: 3, or rather unacceptable: 4, unacceptable: 5, and completely unacceptable: 6), we investigated the qualitative value of the notion of acceptability (i.e., what it means to be acceptable). The interviews were transcribed by anonymizing all the elements, making it possible to recognize the identity of the participants through the name of the club or the age category. The study authors all had a theoretical background in sports-related abuse, and the first and second authors were former elite athletes and coaches, currently serving as performance consultants. The data analysis was performed using thematic content analysis according to Braun and Clarke (2006): (a) familiarization with the data, (b) generation of the initial codes, (c) search for categories, (d) revision of categories, (e) definition and naming of categories, and (f) production of the report. Thus, each interview transcript was read several times and the units of meaning emerging from each interview were highlighted in the text (e.g., “[. . .] as soon as it’s really something that will hurt the athlete”). Then, various subcategories (e.g., well-being) and main categories (e.g., effects) emerged from each interview with several units of meaning (e.g., hurting or harming the athlete) associated in each category (e.g., unacceptable). A summary with the units of meaning, categories, subcategories and main categories in line with the research questions were produced. Finally, a summary table of the units of meaning, main categories and subcategories for all the participants were created. To ensure a more reliable and coherent thematic analysis, the process was followed in parallel by the first two authors. In parallel, based on the quality standards for qualitative research advocated by Smith and McGannon (2018), alternative interpretations of the data were explored. The data were organized into tables to facilitate critical discussion with regard to the study purpose. In parallel, the first author of the study, which has over 15 years of experience as a mental performance and sport consultant, conducted the interviews. Thus, she was able to ask relevant questions and obtain accurate answers to our research questions. To this end, the second author acted as a critical friend, scrutinizing the process of data analysis while challenging and questioning the first author about her experiences, values and beliefs which were essential to minimizing internal bias. Finally, the three authors held several meetings to discuss the analysis, encourage reflection, and stimulate interpretation of the data.

## Results

### Acceptable Abusive Coaching Behaviors

According to Table 2, most abusive coaching behaviors were considered unacceptable by the respondents (i.e., score greater than 3.5). Among the *Contact behaviors*, all behaviors were deemed unacceptable except shaking (*p* = .1189). Among the *Verbal behaviors*, all behaviors were deemed unacceptable except giving a positive opinion about appearance or weight, teasing, giving negative feedback about performance, comparing performance with that of another, and yelling (all tests *p* = 1). Among the *Control behaviors*, all behaviors were considered unacceptable except punishing for bad behavior (*p* = 1), weighing (*p* = 1), and refuse a break during practice (*p* = .06). Among the *Demand behaviors*, more than half the behaviors were considered acceptable, namely asking to start a diet (*p* = .9998), asking to take medication for treatment (*p* = .9984), asking to train despite tiredness (*p* = .9985), asking to participate in a competition despite tiredness (*p* = .9925), asking to perform extremely intense physical activity until exhaustion (*p* = .9589), asking to perform beyond abilities (*p* = .8724), and asking to reach a specific weight (*p* = .8319).

**Table 2.** Results of Tests of Acceptance for a Series of Coaching Behaviors.

	Acceptable	Unacceptable
Contact behaviors	Shake	Catch / grab by the arm^**^ Push or shove^***^ Pinch^***^ Project^***^ Knock down^***^ Throw an object at^***^ Kick^***^ Pull hair^***^ Hit with an object^***^ Slap^***^ Punch^***^ Bite^***^
Verbal behaviors	Give a positive opinion about appearance or weight Tease Give negative feedback about performance Compare performance with that of another Shout at / yell at	Give a negative opinion about appearance or weight^***^ Threaten to stop coaching or kick out^***^ Make fun of in front of other people^***^ Belittle / ridicule^***^ Verbally humiliate^***^ Tell lies about^***^ Threaten to do harm^***^
Control behaviors	Punish for bad behavior Control weight (weighing) Refuse a break during a practice	Throw or hit objects^***^ Punish for not respecting the weight limit^***^ Exclude^***^ Do not select without explanation^***^ Ignore or act detached, indifferent^***^ Deny access to water, or toilets ^***^
Demand behaviors	Ask to adopt a diet Ask to take medication for treatment Ask to train despite tiredness Ask to compete despite tiredness Ask to perform extremely intense physical activity until exhaustion Ask to perform beyond abilities Ask to reach a specific weight	Ask not to talk about what happens in training^***^ Ask to train despite injury^***^ Ask to limit links with social networks^***^ Ask to compete despite injury^***^ Ask to humiliate, ridicule, physically hurt or threaten another athlete^***^

*Note.* Behaviors with *p*-value <.05 are considered unacceptable (^**^p < .01, ^***^p < .001). Details of scores and tests are available on request.

More precisely, athletes’ acceptance of abusive coaching behaviors was different depending on the genders and the countries (see Table 3). Female athletes accepted a higher number of contact, verbal, control, and demand behaviors compared to male athletes (mean, respectively, 2.63, 3.46, 3.05, 3.14 vs. 4.27, 5.31, 5.37, 6.08, *p* < .001). Moreover, French athletes tended to accept more different control behaviors (5.71) than Canadian (3.82) and Swiss (4.04) athletes, whereas Canadian athletes tended to accept a lower number of contact, verbal, control, and demand behaviors (*p* = .043, .002, <.001, <.001, respectively) than Swiss or French athletes. However, there were no significant differences by sport characteristics.

**Table 3.** Descriptive Statistics of Number of Items Accepted (i.e., Score <3.5) Per Category of Coaching Behavior and Test of Difference According to Demographic and Sports Characteristics.

	Contact Behaviors				Verbal Behaviors				Control Behaviors				Demand Behaviors			
	*n*	*M*	*SD*	*P*	*n*	*M*	*SD*	*p*	*n*	*M*	*SD*	*p*	*n*	*M*	*SD*	*p*
Total	355	3.53	4.76		346	4.47	3.09		337	4.33	3.21		329	4.79	3.82	
Male	194	4.27	4.76	<.001	189	5.31	2.87	<.001	186	5.37	2.95	<.001	185	6.08	3.34	<.001
Female	161	2.63	4.62		157	3.46	3.05		151	3.05	3.06		144	3.14	3.77	
Individual	73	3.82	5.24	.488	71	4.32	3.14	.581	69	3.91	3.40	.110	66	5.05	4.07	.679
Team	282	3.45	4.64		275	4.51	3.08		268	4.44	3.15		263	4.73	3.76	
International	26	3.96	4.86	.746	25	4.52	2.81	.365	25	4.72	2.99	.433	25	6.04	3.37	.099
National	110	2.94	4.11		108	4.05	2.70		105	3.99	2.98		103	4.93	3.77	
Reg/Prov	219	3.77	5.04		213	4.68	3.29		207	4.46	3.34		201	4.56	3.89	
Switzerland	209	3.49	4.57	.043	202	4.26	2.90	.002	196	4.04	3.07	<.001	190	4.74	3.74	<.001
Quebec	76	2.79	4.54		74	4.07	3.30		72	3.82	3.29		70	3.56	3.82	
France	70	4.44	5.43		70	5.50	3.20		69	5.71	3.18		69	6.17	3.65	

### Criteria for Acceptance of Abusive Coaching Behaviors

As shown in Table 4, four main categories emerged from the athletes’ comments regarding the acceptance of coaches’ behaviors (i.e., expectations regarding coaches’ role, negative effects, circumstances, and types of behaviors).

**Table 4.** Criteria for Acceptance of Abusive Coaching Behaviors.

Categories		Subcategories	Main Categories	Number of Quotes
Toward unacceptable (coded as unacceptable: 1, 2, 3)	Toward acceptable (coded as acceptable: 4, 5, 6)		Expectations regarding coach’s role	10
Hurting the athlete Putting the athlete down Making the athlete perform less well Affecting physical health Affecting mental health	Make the athlete not respect the rules Make the athlete respect the coach Make the athlete listen Meet the high-performance requirements	Coach intention		
Not constructive for progress Detrimental to the athlete’s performance	Beneficial to the athlete’s performance or success Beneficial for the athlete’s improvement or progress Beneficial for the athlete’s development and growth	Performance and development		
Forcing or imposing on the athlete Not respecting the athlete’s desires and motivations Asking for something the athlete cannot or will not do		Free will		
Not discussing, explaining Not helping the athlete No encouragement	Constructive and explaining	Feedback		
Not treating all athletes the same Targeting one athlete Not liking one athlete		Fairness		
Athlete injured Dangerous for the athlete Not respecting the athlete		Respect		
Affecting the athlete’s mental health Making the athlete feel bad	Beneficial for the athlete’s health	Well-being	Effects	10
Hurting or harming the athlete		Pain		
Affecting desire to train		Motivation		
Bothering the athlete		Discomfort		
Affecting the body		Body		
Scaring the athlete Devaluating the athlete		Mental		
Impacting athlete self-confidence		Self-confidence		
Constantly Regularly Several times	Once From time to time	Frequency	Circumstances	6
Athlete feels powerless Taking advantage of coach’s power Not considering power differences		Power of the coach		
Risky for the athlete	Adjustment of training	Fatigue		
Violent gestures Insult Aggressiveness Meanness		Violent	Type of behaviors	5
Punishing for poor performance Punishing for body issues		Punitive		
Demeaning the athlete Negative words or thoughts		Devaluating		
Entering the athlete’s private life Entering the athlete’s intimacy Interfering with the athlete’s life balance		Intrusive		

First, expectations regarding a coach’s role (i.e., coach intention, athletes’ performance and development, athletes’ free will, feedback given to athletes, fairness, and respect to all athletes) largely explain the acceptance of coaching behaviors. In this category, the coaches’ intention behind the behavior adopted, as well as its justification for the athletes’ performance and development, mostly explained the limit beyond which a coaching behavior became unacceptable. For more than half the athletes interviewed, if the coach's goal was to hurt the athlete, put the athlete down, or make the athlete perform less well, the behavior was described as unacceptable: “From the moment the coach intends to hurt the player, it is unacceptable (Athlete 3),” “When it is something that has an ulterior motive of hurting or injuring the athlete, it’s unacceptable! (Athlete 5)” Conversely, two athletes considered that any behavior aiming to make the athlete respect the coach and the rules or to meet the high-performance demands was consider acceptable. In this perspective, athlete 8 stated that “[. . .] when the coach wants or tries to put you down so that you are less strong psychologically during performance” as unacceptable, whereas athlete 9 explained that “When the athlete crosses the line, does not respect the rules or the coach” any coaching behavior can be acceptable.

Whether the behavior was detrimental or beneficial to the athlete’s performance and progress was the second primary explanation regarding acceptable or unacceptable coaching behavior. For example, “When it is no longer constructive to his progress (athlete 10)” was described as unacceptable whereas “[. . .] anything that aims to improve the athlete and lead him or her to success (athlete 4)” was considered acceptable. Second, coaches’ behaviors were considered unacceptable if they had negative effects on the athlete. These negative effects were organized into seven categories (i.e., well-being, pain, motivation, discomfort, body, mental, self-confidence). The fact that the coach’s behaviors affect the athlete’s mental health, makes the athlete feel bad, or hurt the athlete were the three most frequently cited negative effects of unacceptable coaching behaviors. These results were illustrated by the words of athlete 4: “[From] the moment it starts to hurt the athlete” and athlete 6 “[ . . .] as soon as it’s really something that will have a negative impact on the athlete and that will affect him psychologically [. . .].” Third, behavior frequency determined whether a behavior was acceptable or not: “Screaming a little, once, I think it's acceptable” according to athlete 4, whose words echo those of athlete 6, who explained that “It also depends on the frequency of things that can happen once, but if it happens frequently, it can be quite disturbing for the athlete.” Finally, athletes considered any violent, punitive, devaluating, or intrusive coaching behavior unacceptable. For example, athlete 10 said: “When the coach devalues the athlete, it is unacceptable,” while athlete 9 shared: “If it is violent with insults, it is unacceptable.”

## Discussion

The present study aimed to (a) identify the acceptable abusive coaching behaviors and (b) characterize qualitatively the criteria for the acceptance of abusive coaching behaviors. Our results revealed that these competitive athletes considered almost all contact abusive coaching behaviors (i.e., all except shaking) as unacceptable, whereas several types of others behaviors were considered acceptable (e.g., teasing, shouting at/yelling at, giving negative feedback about performance, controlling athletes’ weight, asking them to reach a specific weight and/or to perform extremely intense physical activity until exhaustion and/or to perform beyond their abilities). The results also underlined that the number of abusive behaviors was the highest for the category *demands* and lower for the *control* and *verbal* categories. This finding was somewhat unexpected, especially considering the relatively high number of abusive behaviors viewed as acceptable when compared to the literature that documented, for example, negative effects of being yelled at (e.g., Stirling & Kerr, 2008a, 2008b, 2009). However, as a whole, our results align with elite sports norms in which some forms of psychological abuse are often perceived as integral to the athlete’s development and success (Gervis & Dunn, 2004; Stirling & Kerr, 2008a, 2008b, 2009). Athletes’ apparent willingness to tolerate abusive coaching behaviors, aligned with these prevailing sport ethics (Coakley & Donnelly, 2009), suggesting that they remain subject to social control mechanisms inherent within the sporting environment. According to the social information processing model, athletes share cognitive schemas regarding the demands for high performance in their sport at the competitive level and what it takes to reach the top level. Based on our results, these shared schemas encompass several types of psychological abuse—such as being shaken, teased, shouted at/yelled at, asked to perform extremely intense physical activity until exhaustion, and asked to perform beyond abilities—that are considered acceptable. The sports ethic transmitted by a slow socialization by their coach over years of interaction could be considered as a shared code of conduct among coaches and their athletes.

Our study also revealed that beyond the abusive coaching behavior itself, the expectations about the role of the coach, the negative effects of coaching behaviors, the circumstances in which the behavior occurs, and the nature of certain behaviors (i.e., abusive, punitive, demeaning, and intrusive) may characterize a coaching behavior as unacceptable. In contrast, the coaching behavior was described as acceptable if it corresponded to positive and constructive coach’s role, athletes’ well-being and specific circumstances of training. These results showed that if it was beneficial for an athlete’s performance, improvement or progress or if there was an adjustment of training, there were enough reasons to accept these abusing coaching behaviors. This rational was based on whether they met the requirements of competitive sport.

These explanations given by the athletes could refer to the situated aspect of coaching behaviors that Jones et al. (2002) described as an opportunistic dynamic social activity “which is inextricably linked to both the constraints and opportunities of human interaction” (p. 35). From this perspective, coaching behaviors should be considered as potentially abusive depending on the multiple characteristics identified in the present study (e.g., coach intention, negative effects, circumstances), but acceptable if they promote athletic development and performance. Beyond shared cognitive schemas, our results suggest that some behaviors can be assimilated by socialization without rational thinking whereas others are subject to the critical eye of the athletes. Social cognitive theory explains this result as follows: “social systems are the product of human activity, and social systems, in turn, help to organize, guide, and regulate human affairs” (Bandura, 2006; p. 165). Since most human functioning is socially situated (Bandura, 2006), a psychological concept like coaching abuse appears to be socially embedded and this might explain why some coaching behaviors are described as acceptable by athletes. Moreover, Bandura (2006) described human agency as encompassing both collective agency, including social groups with shared interests and common goals through sharing experiences, knowledge and skills, and personal agency (i.e., trained individuals becoming more active in their environment and acting on other individuals). This last point could explain the abusive coaching behaviors described as unacceptable by the athletes.

Finally, this study revealed differences in the number of abusive coaching behavior accepted according to participants’ gender and country of residence. Our results revealed that participants from Quebec and female athletes tended to accept less different abusive coaching behaviors than French and Swiss counterparts. This finding could be explained by the fact that cultural values in Quebec are emphasizing a more permissive, inductive approach that excludes forms of coercive control and physical violence in educational settings in which sports are included (Claes et al., 2011; Clément et al., 2019). In contrast, it has been observed that a more punitive approach that respects tradition still exists in France (Suizzo, 2002). Such divergent approaches and philosophies could explain the differences among Quebec athletes by a lack of tolerance for strict or repressive conducts. Moreover, Coaching Association of Canada considers sport as linked to a range of positive outcomes, including improved mental health and well-being and describes coaches as key actors influencing athletes’ mental health (Coaching Association of Canada, 2023). Regarding the gender of athletes, the world of sport can be seen as an arena for men to confront each other, exerting their power and proving their virility (MacArthur & Shields, 2015; Steinfeldt et al., 2012), which might explain why physical abuse is more acceptable to male athletes. While male athletes tend to report more physical abuse than female athletes (e.g., Hauw et al., 2024b; Marsollier et al., 2021; Vertommen et al., 2016), the abuse acceptance could be seen as a risk factor influencing how athletes interpret and ultimately respond to it (Gracia et al., 2020). Conversely, although female athletes tend to report more psychological abuse than men (e.g., Marsollier et al., 2021; Vertommen et al., 2016; Willson et al., 2022), our results suggest that they accept this form of abuse less than male athletes. While this study has revealed an important risk factor of experiencing coach abuse: acceptance, and its relationship with gender and country, more studies are needed to explore the link between abuse acceptance and abuse prevalence.

The present study shows several limitations. First, we examined acceptance of abusive coaching behaviors but not how these perceptions were built across athletes’ careers. In other words, the respondents did not tell their personal stories about sports situations that made them normalize and legitimate abusive behaviors. In this perspective, regarding theories of socialization, our study would have benefited to include the length of sport experience. In addition, the analyses did not focus on perceptions of abusive coaching behaviors depending on success or performance. Yet, as revealed by Marsollier and Hauw (2022) and Stirling and Kerr (2009), experiences of abuse may be dependent on the moment in a given career and athletic outcomes. Future qualitative studies should go further by exploring the dynamic of the experience of coaching behaviors and the coach-athlete interactions that can turn a normal sports situation (e.g., asking an athlete to lose weight to make the category) into an abusive one (lack of support leading to eating disorders, see Marsollier & Hauw, 2022). Finally, the small sample size and voluntary sample must be mentioned as limitations. While many gender differences were observed, the distribution of age, country and level of practice varied by gender. It would therefore have been good to control several variables (i.e., analyze by gender and country, gender, and level of sport), but the sample size did not allow us to conduct analyses in such subgroups.

In summary, our study contributes to the literature by furthering the understanding of what underlies these abusive behaviors in sport and their acceptance. Ultimately, we believe this study can help athletes and coaches to develop a keener appreciation of exactly which coaching behaviors can be used to enhance athletes’ performance. Thus, any violent, punitive, devaluating, or intrusive coach behavior that negatively affects athletes’ performance or well-being should be avoided. To conclude, sports-related abuse is a complex phenomenon that needs to be explained and denounced, and one approach is to examine the perceptions of the victims. This study refines the conception of what is acceptable or not in the field of coaching behaviors, with the hope that the elite sports community will have a keener appreciation of what constitutes a more respectful approach to preserving human dignity and rights in the sports arena.
